# Supplementary material for: NGC1818 unveils the origin of the extended main-sequence turn-off in young Magellanic Clouds clusters
Source: Nat Commun. 2022 Jul 26;13:4325. doi: 10.1038/s41467-022-31977-y (PMC9325706; doi:10.1038/s41467-022-31977-y)
Supplement: Supplementary file 2 — Description of Additional Supplementary Files [file 41467_2022_31977_MOESM2_ESM.pdf]

Description of Additional Supplementary Files

File name: Supplementary Data 1
Description: the file contains the photometric catalog generated in this work, and exploited to analyze NGC1818. It contains the following columns:

col 1 = x [pixel]
col 2 = y [pixel]
col 3 = f3 [F336W magnitude]
col 4 = self3 [0=Bad star, 1=Good star]
col 5 = f6 [F6206W magnitude]
col 6 = self6 [0=Bad star, 1=Good star]
col 7 = f8 [F814W magnitude]
col 8 = self8 [0=Bad star, 1=Good star]
col 9 = cl [0= star not in cluster field, 1= star in cluster field]
col 10 = fl [0= star not in reference field, 1= star in reference field]
coll 11 = radius [distance in pixel from cluster center (Xc, Yc)=(4574.3466, 4523.6471)]
